# Stroke aetiological classification reliability and effect on trial sample size: systematic review, meta-analysis and statistical modelling

**DOI:** 10.1186/s13063-019-3222-x

**Published:** 2019-02-08

**Authors:** Azmil H. Abdul-Rahim, David Alexander Dickie, Johann R. Selvarajah, Kennedy R. Lees, Terence J. Quinn, On behalf of VISTA Collaborators, K. R. Lees, A. Alexandrov, P. M. Bath, E. Berge, E. Bluhmki, N. Bornstein, C. Chen, L. Claesson, S. M. Davis, G. Donnan, H. C. Diener, M. Fisher, M. Ginsberg, B. Gregson, J. Grotta, W. Hacke, M. G. Hennerici, M. Hommel, M. Kaste, P. Lyden, J. Marler, K. Muir, N. Venketasubramanian, R. Sacco, A. Shuaib, P. Teal, N. G. Wahlgren, S. Warach, C. Weimar

**Affiliations:** 1 Institute of Neuroscience and Psychology, University of Glasgow, Room 0.07, Office Block, Queen Elizabeth University Hospital, G51 4TF, Glasgow, UK; 2 Institute of Cardiovascular and Medical Sciences, University of Glasgow, Glasgow, UK; 3 Department of Neurology, Institute of Neurological Sciences, Queen Elizabeth University Hospital, Glasgow, UK

**Keywords:** Stroke, Classification, Aetiology

## Abstract

**Background:**

Inter-observer variability in stroke aetiological classification may have an effect on trial power and estimation of treatment effect. We modelled the effect of misclassification on required sample size in a hypothetical cardioembolic (CE) stroke trial.

**Methods:**

We performed a systematic review to quantify the reliability (inter-observer variability) of various stroke aetiological classification systems. We then modelled the effect of this misclassification in a hypothetical trial of anticoagulant in CE stroke contaminated by patients with non-cardioembolic (non-CE) stroke aetiology. Rates of misclassification were based on the summary reliability estimates from our systematic review. We randomly sampled data from previous acute trials in CE and non-CE participants, using the Virtual International Stroke Trials Archive. We used bootstrapping to model the effect of varying misclassification rates on sample size required to detect a between-group treatment effect across 5000 permutations. We described outcomes in terms of survival and stroke recurrence censored at 90 days.

**Results:**

From 4655 titles, we found 14 articles describing three stroke classification systems. The inter-observer reliability of the classification systems varied from ‘fair’ to ‘very good’ and suggested misclassification rates of 5% and 20% for our modelling. The hypothetical trial, with 80% power and alpha 0.05, was able to show a difference in survival between anticoagulant and antiplatelet in CE with a sample size of 198 in both trial arms. Contamination of both arms with 5% misclassified participants inflated the required sample size to 237 and with 20% misclassification inflated the required sample size to 352, for equivalent trial power. For an outcome of stroke recurrence using the same data, base-case estimated sample size for 80% power and alpha 0.05 was *n* = 502 in each arm, increasing to 605 at 5% contamination and 973 at 20% contamination.

**Conclusions:**

Stroke aetiological classification systems suffer from inter-observer variability, and the resulting misclassification may limit trial power.

**Trial registration:**

Protocol available at reviewregistry540.

**Electronic supplementary material:**

The online version of this article (10.1186/s13063-019-3222-x) contains supplementary material, which is available to authorized users.

## Background

Stroke is a syndrome with heterogeneous aetiologies. Grouping these aetiologies together has been effective when developing acute interventions such as intravenous thrombolysis, but improved access to imaging, rhythm monitoring and biomarkers may support a more individualised approach to treatment and research. We increasingly acknowledge the relevance of aetiology as a determinant of prognosis, as a risk factor for recurrence and as a potential treatment effect moderator.

Various classification systems have been developed to define stroke aetiology using clinical features and the results of ancillary investigations. These aetiological classification tools attempt to categorise stroke, and the subtypes usually include cardioembolism, large vessel atheroma and small vessel disease. Robust classification of aetiology is essential to guide treatment decisions when the optimal pharmacological treatment differs between aetiological groups. This situation is seen with prevention of cardioembolic (CE) versus large vessel stroke, where the differing pathologies require differing treatment strategies.

The reliability of a classification tool is a measure of the degree to which results are reproducible when repeated observations are made, either by the same physician on repeated assessments (intra-observer reliability) or between physicians (inter-observer reliability). Several factors may impair classification reliability, including inherent properties of the classification system itself (such as its complexity), properties of the patient population (such as the spectrum of disease aetiology), the quality and completeness of ancillary data and the expertise of the physicians using the classification algorithm.

Impaired reliability, regardless of cause, will result in misclassification error. This misclassification is problematic for a number of reasons. It will lead to potentially biased estimates of the prevalence of disease aetiologies and misdirected treatment decisions. Misclassification error may compromise the statistical power and efficiency of research studies, inflating their costs and reducing sensitivity. Finally, misclassification may undermine the estimate of effect size and reduce apparent efficacy and cost effectiveness [1, 2].

All of these issues are particularly pertinent to the emerging literature on embolic stroke of undetermined source (ESUS) [3, 4]. Ongoing and completed large trials in this area were based on a trial entry classification paradigm where patients with atrial fibrillation (AF) or ESUS receive anticoagulation and patients with other, non-cardioembolic (non-CE) aetiologies receive antiplatelet treatment. Baseline misclassification of stroke subtype and subsequent misallocation to treatment arms could compromise the power of these trials to demonstrate utility. For example, if a patient with an arterial atheromatous cause of stroke is erroneously recruited into an ESUS treatment arm, they will not benefit from the treatment given, and this will dilute the power to see a treatment effect.

We designed a programme of work to model the potential effect of aetiological misclassification on sample size in a hypothetical trial of anticoagulant in CE stroke. We first describe the reliability (inter-observer variability) of stroke classification systems and then model the effect of this misclassification on sample size required to show the effect of chosen treatments in terms of survival and stroke recurrence.

## Methods

We used an iterative approach to our analyses. First, we performed a systematic review and meta-analysis to quantify potential misclassification rates across different stroke classification systems. We then performed a scoping analysis, using aggregate data from published trials to estimate the potential effect of misclassification. Finally, we used individual patient-level data to model the impact of misclassification on a hypothetical trial.

### Systematic review

We followed, where appropriate, Preferred Reporting Items for Systematic Review and Meta-Analyses (PRISMA) best practice guidance for design, conduct and reporting of our systematic review. We worked according to a pre-defined protocol (Research Registry Unique Identifying Number reviewregistry540, accessible on URL https://www.researchregistry.com/). Our primary aim for the systematic review was to describe the inter-observer reliability of stroke classification systems. The metric of interest was reliability of the classification tool, i.e. agreement between observers. We did not pre-define the classification scales of interest.

We devised a sensitive search strategy using validated search terms across multidisciplinary electronic databases, from database inception to December 2018 inclusive (Additional file 1: Table S1). We used citation searching (backwards searching) and assessed all articles that had cited the index article (forward searching). There were no restrictions relating to date of publication, the number of participants or assessors. Only papers published in peer-reviewed, English language scientific journals were considered.

Title and abstracts generated from the electronic database searches were screened for relevance, irrelevant titles and abstracts were excluded and full-text articles were inspected to determine eligibility. Data from studies meeting our inclusion criteria were extracted to a proforma. All aspects of the title searching, assessment and data extraction were performed by two independent researchers. Decisions were made by consensus with recourse to a third arbitrator as necessary. No authors were contacted for the study.

We assessed risk of bias using Guidelines for Reporting Reliability and Agreement Studies (GRRAS) [5]. The main characteristics analysed were stroke classification system, stroke population, assessor population, sample size calculation, sampling methods, blinding and reporting of reliability with a corresponding measure of uncertainty.

We described inter-observer reliability using the kappa (κ) statistic, using standard definitions of poor (κ = 0.00–0.20), fair (κ = 0.21–0.40), moderate (κ = 0.41–0.60), good (κ = 0.61–0.80) and very good (κ = 0.81–1.00) reliability [6, 7]. We described the reliability of stroke classification systems and, where data were available, the reliability of classification into CE and non-CE aetiologies.

We used the summary data from the systematic reviews to inform estimates of potential rates of misclassification in a trial that recruited based on stroke aetiology. We used ‘best case’ and ‘worst case’ reliability estimates from the systematic review. There is no agreed approach for converting kappa values (our summary agreement metric from meta-analysis) to a measure of percentage agreement/disagreement (the measure needed for our modelling exercise). We used a conversion that has been previously described [8]. We opted for cautious estimates of misclassification (disagreement) based on the conversion (5% and 20%). We ensured that these values were broadly in keeping with the data presented in papers that describe reliability in terms of percentage agreement as well as kappa.

### Effect of stroke classification on trial sample size: aggregate analyses

**Fig. 1:**
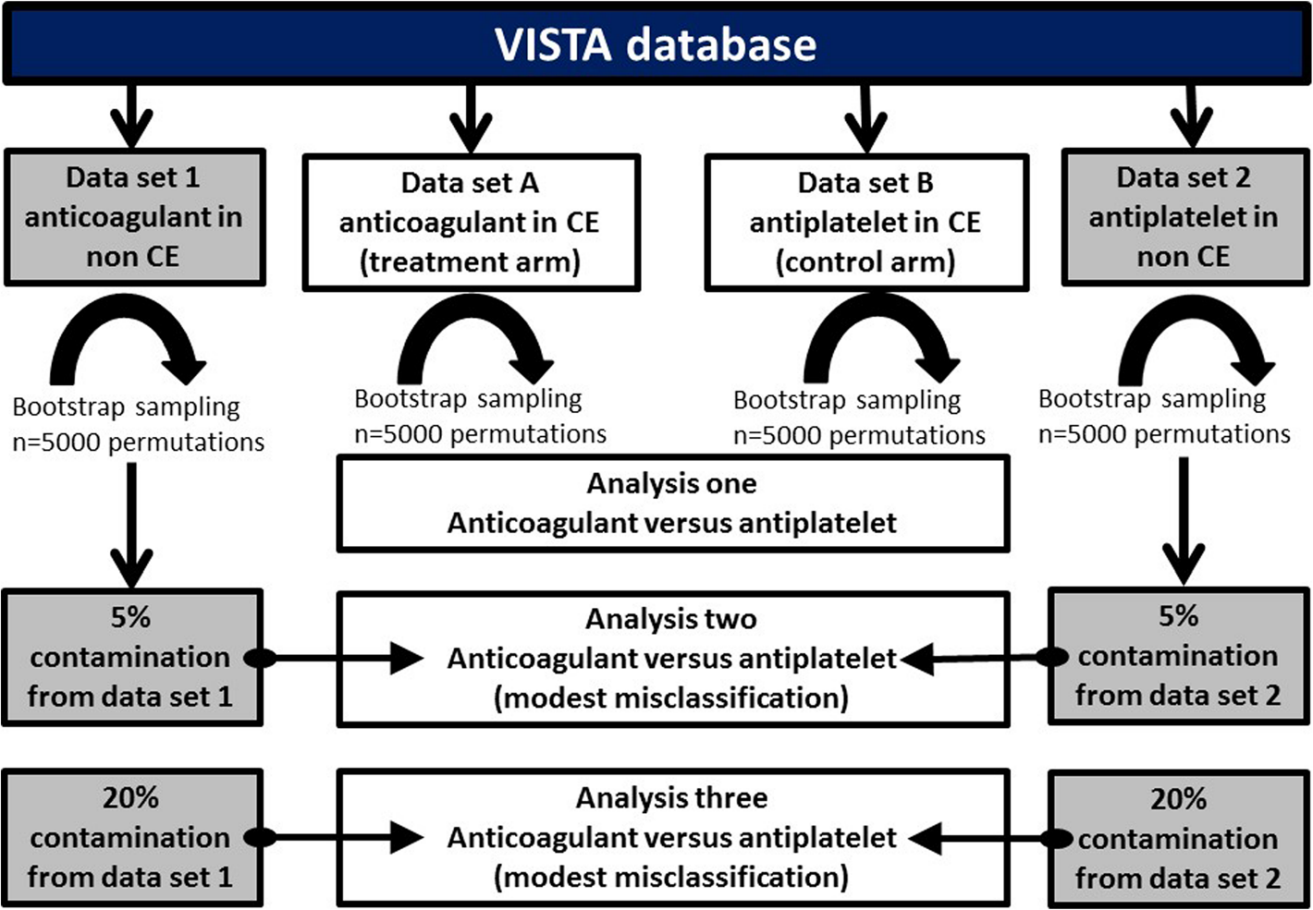
Effect of stroke classification on trial sample size: aggregate analyses. *VISTA* Virtual International Stroke Trials Archive, *CE* cardioembolic

We used our summary estimates of misclassification to inform a series of statistical models. As an initial scoping exercise we created a hypothetical study of anticoagulant versus antiplatelet in CE stroke. We used survival outcomes data from historical stroke trials that included the use of anticoagulant and antiplatelet treatment in CE and non-CE stroke, to give proportional survival in each [9, 10]. We then replaced a proportion of the CE patients with non-CE patients in each treatment arm. We used the Pearson chi-square test for proportion difference, first assuming perfect classification rates and then factoring in differing rates of misclassification. For a given rate of misclassification, we substituted that proportion of non-CE stroke patients into the CE treatment arm and vice versa (Fig. 1).

### Effect of stroke classification on trial sample size: individual patient-level analyses

We then explored the effect of misclassification on a hypothetical trial involving patients with CE stroke using individual patient-level data. We used data from the Virtual International Stroke Trials Archive (VISTA), http://www.virtualtrialsarchives.org/vista/, as the base-case data to inform our models. VISTA is a not-for-profit repository for stroke trial data, containing study quality, anonymised, individual patient-level data on thousands of participants [11, 12]. These data have been used to investigate novel hypotheses, including analyses of stroke assessment scale properties [13, 14].

We tested a hypothetical misclassification scenario; a trial that assesses the efficacy of an oral anticoagulant versus antiplatelet in patients with CE stroke contaminated by patients with non-CE stroke (aetiological misclassification).

From VISTA, we selected populations of CE stroke treated with anticoagulant or antiplatelet agent, and populations of non-CE stroke treated with anticoagulant or antiplatelet. We assumed that patients with known AF and neither large nor small vessel disease were CE. We assumed that patients with no AF and proven large or small vessel disease aetiology of stroke were non-CE. We calculated an initial sample size for outcomes of death and stroke recurrence using aggregate VISTA data from CE-anticoagulant and CE-antiplatelet groups.

We then used bootstrapping simulations with random repetition sampling to create models to our specified sample size, *n* = 7000, using the RAND function in Matrix Laboratory (MATLAB) software. In the hypothetical ‘treatment’ arm, we randomly selected the relevant number of correctly classified patients from the CE cohort treated with anticoagulation and then randomly selected the corresponding number of incorrectly classified patients from the non-CE cohort treated with anticoagulant. We created a ‘control’ arm using the same process, sampling from CE treated with antiplatelet and contaminating with non-CE treated with antiplatelet. We summarised outcomes across 5000 permutations.

All analyses were performed using MATLAB Statistics and Machine Learning Toolbox R2014a (© 1994–2014 The MathWorks, Inc., Natick, MA, USA).

## Results

### Systematic review

From 4655 titles, we found 14 titles that met the inclusion criteria [15–28] (PRISMA diagram, Additional file 1: Figure S1). The included articles described principally three stroke classification systems:

1. The automated Causative Classification of Stroke System (CCS), (*n* = 4 papers) [17, 26–28]
2. The Trial of Org 10172 in Acute Stroke Treatment (TOAST) algorithm, (*n* = 10 papers) [15–24]
3. ASCO system (A for atherosclerosis, S for small vessel disease, C for cardiac source and O for other cause, *n* = 4 papers) [23–26]

Four papers described the reliability of more than one stroke classification system [17, 23, 24, 26]. Four articles were suitable to perform a meta-analysis for ’classic’ TOAST [18–21] and four for CCS [17, 26–28] (Table 1). The assessment of quality and risk of bias are described in Additional file 1: Table S2. Included papers were assessed as low risk of basis for most items except calculation of sample size (not described in 11/14 included papers), blinding of assessors (not described in 9/14 included papers) and quantifying error in reliability estimates (not described in 8/14 papers).

**Table 1:** Characteristics of included studies

Study	Subject no.	Assessors	Assessor no.	Country	Stroke classification	Single site
Inter-rater reliability studies						
Arsava et al., 2010 [27]	50	Various physicians^a^	15	International^d^	CCS	No
Ay et al., 2005 [17]	50	Neurologists	2	USA	CCS/TOAST	No
Ay et al., 2007 [28]	50	Neurologists	5	USA	CCS	No
Chen et al., 2013 [25]	419	Stroke neurologists	2	China	ASCO	Yes
Fure et al., 2005 [16]	38	Junior registrars, experienced physicians	4	Norway	TOAST	Yes
Goldstein et al., 2001 [19]	14	Neurologists, internists	4	USA	TOAST computerised algorithm/ TOAST	No
Gordon et al., 1993 [22]	18	Neurologists	24	USA	TOAST	Yes
Han et al., 2007 [15]	200	Neurologists	3	Korea	Han et al., 2007/TOAST	Yes
Marnane et al., 2010 [26]	381	Physicians^b^	U	Ireland	ASCO/CCS	Yes
Meschia et al., 2006 [20]	30	Neurologists	6	USA	TOAST	No
Nam et al., 2012 [21]	70	Residents, stroke experts	7	Korea	iTOAST/ TOAST	Yes
Selvarajah et al.,2009 [18]	90	Various stroke physicians^c^	4	UK	TOAST	Yes
Wolf et al., 2012 [23]	103	Stroke physician	2	Germany	ASCO/ TOAST	Yes
Intra-rater reliability studies						
Cotter et al., 2011 [24]	106	U	U	UK	ASCO/ TOAST	Yes

All studies were retrospective reviews of clinical materials

*U* unknown, *CCS* Causative Classification of Stroke System, *TOAST* Trial of Org 10172 in Acute Stroke Treatment, *ASCO* atherosclerosis, small vessel disease, cardiac source

^a^Included stroke neurologists, clinical neuroscientist, stroke fellowship, trained emergency physician, neurology resident

^b^Included trained stroke physician, nonstroke specialist

^c^Included senior lecturer and research fellow in stroke medicine, clinical consultant neurologist, senior house officer in clinical neurology

^d^Included USA, Italy, Spain, Germany, Austria, Sweden, UK, Nigeria

We were able to describe reliability for classification scales in general and at the level of individual aetiology. For the different TOAST classification systems, study-level inter-observer reliability varied from ‘fair’ to ‘very good’ (Table 2). Across the eight studies where data were suitable for pooled analysis, overall kappa was moderate (κ = 0.53; 95% confidence interval [CI] 0.49–0.56). For the ’classic’ version of TOAST, pooled reliability was also moderate (κ = 0.55; 95% CI 0.51–0.59) (Additional file 1: Figures S2 and S3).

**Table 2:** Inter-observer reliability of different types of stroke classification systems

Study	Classification system	κ	95% CI
TOAST classification system			
Ay et al., 2005 [17] (*n* = 50)	TOAST	0.78	0.64–0.92
Fure et al., 2005 [16] (*n* = 38)	TOAST	0.30	*
Goldstein et al., 2001a [19] (*n* = 14)	TOAST (11 categories)	0.29	0.21–0.37
	TOAST	0.42	0.32–0.53
Goldstein et al., 2001b [19] (*n* = 20)	TOAST computerised algorithm	0.68	0.44–0.91
Gordon et al., 1993 [22] (*n* = 18)	TOAST	0.64	*
Han et al., 2007 [15] (*n* = 200)	TOAST	0.78	*
Meschia et al., 2006 [20] (*n* = 30)	TOAST	0.54	0.48–0.60
Nam et al., 2012 [21] (*n* = 70)	iTOAST	0.79	0.71–0.87
	TOAST	0.69	0.60–0.78
Selvarajah et al., 2009 [18] (*n* = 90)	TOAST	0.42	0.32–0.52**
Wolf et al., 2012 [23] (*n* = 103)	TOAST	0.95	*
CCS			
Arsava et al., 2010 [27] (*n* = 50)	CCS: 5 subtype	0.80	0.78–0.81
	8 subtype	0.79	0.77–0.80
	16 subtype	0.70	0.69–0.71
Ay et al., 2005 [17] (*n* = 50)	5 subtype	0.86	0.76–0.96
Ay et al., 2007 [28] (*n* = 50)	5 subtype	0.86	0.81–0.91
	8 subtype	0.85	0.80–0.89
	16 subtype	0.80	0.76–0.83
Marnane et al., 2010 [26] (*n* = 381)	5 subtype	0.64	0.44–0.82**
ASCO classification system			
Chen et al., 2013 [25] (*n* = 419)	Phenotype: *A*	0.79	0.74–0.83
	*S*	0.80	0.75–0.85
	*C*	0.87	0.83–0.91
	*O*	0.86	0.78–0.94
Marnane et al., 2010 [26] (*n* = 381)	*A*	0.79	*
	*S*	0.48	*
	*C*	0.88	*
	*O*	0.66	*
Wolf et al., 2012 [23] (*n* = 103)	*A*	0.95	*
	*S*	0.95	*
	*C*	1.00	*
	*O*	0.92	*
Other classification system			
Han et al., 2007 [15] (*n* = 200)	Han et al. (2007) classification	0.82	*

*CI* confidence interval, *TOAST* Trial of Org 10172 in Acute Stroke Treatment, *CCS* Causative Classification of Stroke System, *ASCO* atherosclerosis, small vessel disease, cardiac source

*Unknown 95% CI

**Estimates provided by author

For the different subtypes of CCS classifications, study-level inter-observer reliability ranged from ‘good’ to ‘very good’. The inter-observer reliability of different subtypes of CCS suggested that 5 subtype CSS and 8 subtype CCS had very good overall reliability and 16 subtype CCS had good overall reliability (Table 2). Overall reliability for 5 major CCS subtype was good (κ = 0.81; 95% CI 0.80–0.83) (Table 2 and Additional file 1: Figures S4 and S5).

In the case of ASCO, inter-observer variability was described according to each potential phenotype and varied from perfect (κ = 1) for the ’C’ (cardiac) phenotype to good (κ = 0.66) for the ’O’ (other) phenotype (Table 2).

Based on these summary reliability measures, we estimated proportional misclassification extremes at 5% and 20%, representing the approximate misclassification that may be seen with the most favourable (seen with CCS and ASCO systems) and the least favourable (TOAST) reliability estimates.

### Effect of stroke classification on trial sample size: aggregate analyses

In our initial scoping analyses, using aggregate data from historical trials of anticoagulant in CE and non-CE, we started with a zero misclassification, base-case scenario trial of *n* = 392 to detect an outcome difference in treatment effect in terms of survival (power = 0.8, alpha 0.05). With a misclassification of 5% the required sample size to demonstrate the same effect was *n* = 444 (13% increase). With a misclassification of 20% the required sample size to demonstrate the same effect was *n* = 663 (69% increase). Detailed analysis results are included in Additional file 1: Supplemental Results.

### Effect of stroke classification on trial sample size: individual patient-level analyses

We obtained data for 2066 patients with acute ischaemic stroke from VISTA, of whom 514 had CE on baseline assessment (*n* = 207, 40% were on antiplatelet, the remainder anticoagulant) and 1545 patients with non-CE (*n* = 1171, 76% were on antiplatelet treatment, the remainder anticoagulant). The baseline characteristics of these patients are shown in Additional file 1: Table S3.

For an outcome of death at first follow-up, based on proportions seen in the aggregate data, a base-case estimate was of a required sample size of *n* = 198 for both arms of the trial to detect a between-group difference (power = 0.8, alpha 0.05). The required sample size to demonstrate a statistically significant treatment effect increased to *n* = 237 in each arm (20% increase) at 5% misclassification and *n* = 352 (78% increase) at 20% misclassification. For an outcome of stroke recurrence using these same data, the base-case estimate sample size was *n* = 502, increasing to 605 (21% increase) at 5% contamination and 973 (94% increase) at 20% contamination for each arm of the trial (Table 3).

**Table 3:** Effect of misclassification levels on the required sample size in a hypothetical trial of anticoagulant versus antiplatelet in patients with cardioembolic stroke to demonstrate statistically significant result for the relevant outcomes, using *N* = 5000 permutations

Stroke aetiology misclassification level	Death	Recurrent stroke
0%	198	502
5%	237 (20% increase)	605 (21% increase)
20%	352 (78% increase)	973 (94% increase)

Sample sizes calculated with power = 0.8, alpha = 0.05

A 20% contamination of patients with non-CE stroke in a hypothetical trial of anticoagulant versus antiplatelet treatment in patients with CE stroke would underestimate the effect of anticoagulant treatment by at least 10% for the outcome of death and 15% for the outcome of recurrent stroke, compared to a trial without any contamination. (See Additional file 1: Supplemental Results.)

## Discussion

Our analyses have confirmed that even advanced classification systems for stroke aetiology harbour residual inter-observer variability of at least 5% and potentially much greater. Based on this variability in classification, we have shown that the resulting misclassification contributes a sample size penalty of at least 20% and potential incorrect estimation of the treatment effect size by at least 10%.

It seems plausible that stroke trials targeted at a particular aetiological subgroup may have been underpowered to demonstrate a treatment effect. To take a high-profile example, the New Approach riVaroxaban Inhibition of Factor Xa in a Global trial versus Aspirin to prevenT Embolism in Embolic Stroke of Undetermined Source (NAVIGATE-ESUS) study was terminated early because of futility and excess bleeding on rivaroxaban [4]. One can speculate whether this neutral result was at least partly due to the study being underpowered as a result of baseline misclassification.

Our results align with previous research looking at the effect of misclassification of treatment outcomes in stroke trials [1]. The modified Rankin Scale (mRS) is the most commonly used outcome measure in stroke research [29]. Typically, mRS assessment is based on a clinician’s rating of a patient interview, and inter-observer variability is common [30]. Meta-analysis suggests that mRS assessments have an overall reliability of κ = 0.62 (weighted κ = 0.9) [30], but this may be less (κ = 0.25) in multicentre studies [31]. However, increasing the reliability of the mRS assessments by central adjudication (including across international centres) has been shown to significantly reduce the required trial sample size and to increase trial power [1]. Given the substantial and increasing per patient cost of randomisation into a clinical trial, funders, trialists and industry have been keen to limit the potential effect of misclassification on required sample size. Training in mRS assessment is now mandatory for many trials, and several contemporary international stroke trials are using a system of central expert adjudication of mRS assessments [32–35]. Comparable approaches could be employed to limit aetiological misclassification, with the anticipation of greater trial efficiency. There are many other aspects of trials within stroke and in other disease areas where misclassification could compromise power. More research around the properties of assessment tools could be useful to help us understand the potential impact on study results. Research in stroke suggests that poor reliability is not inherent and some assessment tools have greater reliability than others [36].

### Strengths and limitations of the study

We present a novel analysis on an increasingly pertinent methodological issue in clinical trials. Our estimates of misclassification are based on a comprehensive review of the literature following best practice in systematic review and meta-analysis. Our modelling was based on individual patient-level data from completed clinical trials.

There are limitations to this analysis. Our misclassification modelling analysis makes a number of assumptions. For example, we assume that the historical event data are still relevant to contemporary patients with acute stroke. We also assume ‘perfect’ aetiological classification within the VISTA data that inform our modelling. Converting kappa values from our meta-analyses to rates of misclassification comes with certain caveats. We used an accepted ‘rule of thumb’ [8], but this conversion is imperfect. Based on these criteria, TOAST agreement could be anything from 30 to 80%. We opted for cautious estimates of misclassification (disagreement), although arguably our misclassification rates could have been much higher. In all our analyses, we assume that the misclassification described and modelled above affects the cardioembolic (CE) and the non-CE groups. Finally, it is possible that misclassification may affect one treatment arm more than the other. These limitations are all likely to underestimate any deleterious effects of misclassification on sample size, and so we believe our message remains valid.

Our analysis modelled outcomes of survival and stroke recurrence, as these are the endpoints most commonly described in anticoagulant trials. We acknowledged that other outcomes may also be relevant and subject to misclassification effects, for example functional recovery or non-fatal adverse events.

We use anticoagulation in the context of AF as a model, as these were the data available. It would be unwise to directly extrapolate these data to ESUS trials. The natural history, effect sizes and adverse event risks will differ between ESUS and the proven AF used in our models, but our analysis is designed to be illustrative of the potential statistical effect rather than a direct comment on ESUS trials.

### Implications for future practice and research

The stroke classification systems we studied are imperfect, but defining an underlying aetiology for stroke may still be important for personalised stroke treatment and research. Our findings should not deter clinicians and trialists from trying to classify patients; rather, we believe that strategies to improve the reliability of aetiological classification are needed. Some may argue that the move towards precision in therapeutics is less relevant in stroke, as recurrence is not unique to the aetiology of the index event. In the secondary stroke prevention subgroups of the non-vitamin K antagonist anticoagulant trials, 50% of the patients in addition to AF had atherosclerosis in terms of stable coronary artery disease, peripheral artery disease or plaques in the carotid arteries [37–39]. In addition, we know from the long-term electrocardiographic (ECG) monitoring trials that 10–15% of patients per year develop silent paroxysmal AF [40, 41]. However, trials of aetiologically specific intervention continue, and it seems sensible to minimise and account for the effect of any aetiological misclassification. Perhaps future trials should have the aetiological classification done by independent physicians at study entry, halfway through the study and at the end of the study.

Previous work on mRS assessments showed that introduction of training, structured assessment and consensus review can reduce misclassification in outcome adjudication [1]. It seems plausible that the same may be true for aetiological classification. Further work to quantify misclassification in contemporary stroke practice is needed. Our work offers some rough estimates of misclassification effect that stroke trialists could factor into the design of trials and estimates of required sample size.

## Conclusions

Aetiological classification systems are associated with inter-observer variability. The resulting misclassification of stroke aetiology may reduce trial power to adequately identify effective stroke prevention therapy, reduce the effect size and increase associated trial costs.

## Additional file

Additional file 1: **Table S1.** Search strategy. **Table S2.** Reporting quality and risk of bias assessment of the studies. **Table S3.** Baseline characteristics of the VISTA cohort used to analyse the effect of stroke classification on trial sample size. **Figure S1.** PRISMA 2009 flow diagram: literature search strategy. **Figure S2.** Forest plot describing inter-observer reliability (κ) across studies with different versions of TOAST. *CI* confidence interval. **Figure S3.** Forest plot describing inter-observer reliability (κ) across the studies for classic version of TOAST. *CI* confidence interval. **Figure S4.** Forest plot comparing inter-observer reliability (κ) across the studies for CCS subtypes. *CI* confidence interval. **Figure S5.** Forest plot describing inter-observer reliability (κ) across studies for CCS 5 subtype. *CI* confidence interval. **Supplemental Results.** Description of aggregate analysis using VISTA cohort. (DOCX 210 kb)

## Abbreviations

AF: Atrial fibrillation
CE: Cardioembolic
ECG: Electrocardiographic
ESUS: Embolic stroke of undetermined source
mRS: Modified Rankin Scale
NAVIGATE-ESUS: New Approach riVaroxaban Inhibition of Factor Xa in a Global trial versus Aspirin to prevenT Embolism in Embolic Stroke of Undetermined Source (trial)
VISTA: Virtual International Stroke Trials Archive

## References

[1] McArthur KS, Johnson PC, Quinn TJ, Higgins P, Langhorne P, Walters MR, Weir CJ, Dawson J, Lees KR (2013). Improving the efficiency of stroke trials: feasibility and efficacy of group adjudication of functional end points. Stroke.

[2] Muller MJ, Szegedi A (2002). Effects of interrater reliability of psychopathologic assessment on power and sample size calculations in clinical trials. J Clin Psychopharmacol.

[3] Diener HC, Easton JD, Granger CB, Cronin L, Duffy C, Cotton D, Brueckmann M, Sacco RL (2015). Design of Randomized, double-blind, Evaluation in secondary Stroke Prevention comparing the EfficaCy and safety of the oral Thrombin inhibitor dabigatran etexilate vs. acetylsalicylic acid in patients with Embolic Stroke of Undetermined Source (RE-SPECT ESUS). Int J Stroke.

[4] Hart RG, Sharma M, Mundl H, Kasner SE, Bangdiwala SI, Berkowitz SD, Swaminathan B, Lavados P, Wang Y, Wang Y, Davalos A, Shamalov N, Mikulik R, Cunha L, Lindgren A, Arauz A, Lang W, Czlonkowska A, Eckstein J, Gagliardi RJ, Amarenco P, Ameriso SF, Tatlisumak T, Veltkamp R, Hankey GJ, Toni D, Bereczki D, Uchiyama S, Ntaios G, Yoon B-W, Brouns R, Endres M, Muir KW, Bornstein N, Ozturk S, O’Donnell MJ, De Vries Basson MM, Pare G, Pater C, Kirsch B, Sheridan P, Peters G, Weitz JI, Peacock WF, Shoamanesh A, Benavente OR, Joyner C, Themeles E, Connolly SJ (2018). Rivaroxaban for stroke prevention after embolic stroke of undetermined source. N Engl J Med.

[5] Kottner J, Audige L, Brorson S, Donner A, Gajewski BJ, Hrobjartsson A, Roberts C, Shoukri M, Streiner DL (2011). Guidelines for Reporting Reliability and Agreement Studies (GRRAS) were proposed. J Clin Epidemiol.

[6] McGinn T, Wyer PC, Newman TB, Keitz S, Leipzig R, Guyat G (2004). Tips for learners of evidence-based medicine: 3. Measures of observer variability (kappa statistic). CMAJ.

[7] Landis JR, Koch GG (1977). The measurement of observer agreement for categorical data. Biometrics.

[8] McHugh ML (2012). Interrater reliability: the kappa statistic. Biochemia medica.

[9] Hylek EM, Go AS, Chang Y, Jensvold NG, Henault LE, Selby JV, Singer DE (2003). Effect of intensity of oral anticoagulation on stroke severity and mortality in atrial fibrillation. N Engl J Med.

[10] Rothwell PM, Algra A, Chen Z, Diener HC, Norrving B, Mehta Z (2016). Effects of aspirin on risk and severity of early recurrent stroke after transient ischaemic attack and ischaemic stroke: time-course analysis of randomised trials. Lancet.

[11] Ali M, Bath P, Brady M, Davis S, Diener HC, Donnan G, Fisher M, Hacke W, Hanley DF, Luby M, Tsivgoulis G, Wahlgren N, Warach S, Lees KR, Comm VS (2012). Development, expansion, and use of a stroke clinical trials resource for novel exploratory analyses. Int J Stroke.

[12] Ali M, Bath PM, Curram J, Davis SM, Diener HC, Donnan GA, Fisher M, Gregson BA, Grotta J, Hacke W, Hennerici MG, Hommel M, Kaste M, Marler JR, Sacco RL, Teal P, Wahlgren NG, Warach S, Weir CJ, Lees KR (2007). The Virtual International Stroke Trials Archive. Stroke.

[13] Ali M, Fulton R, Quinn T, Brady M (2013). How well do standard stroke outcome measures reflect quality of life? A retrospective analysis of clinical trial data. Stroke.

[14] MacIsaac RL, Ali M, Taylor-Rowan M, Rodgers H, Lees KR, Quinn TJ (2017). Use of a 3-item short-form version of the Barthel Index for use in stroke. Stroke.

[15] Han SW, Kim SH, Lee JY, Chu CK, Yang JH, Shin HY, Nam HS, Lee BI, Heo JH (2007). A new subtype classification of ischemic stroke based on treatment and etiologic mechanism. Eur Neurol.

[16] Fure B, Wyller TB, Thommessen B (2005). TOAST criteria applied in acute ischemic stroke. Acta Neurol Scand.

[17] Ay H, Furie KL, Singhal A, Smith WS, Sorensen AG, Koroshetz WJ (2005). An evidence-based causative classification system for acute ischemic stroke. Ann Neurol.

[18] Selvarajah JR, Glaves M, Wainwright J, Jha A, Vail A, Tyrrell PJ (2009). Classification of minor stroke: intra- and inter-observer reliability. Cerebrovasc Dis.

[19] Goldstein LB, Jones MR, Matchar DB, Edwards LJ, Hoff J, Chilukuri V, Armstrong SB, Horner RD (2001). Improving the reliability of stroke subgroup classification using the Trial of ORG 10172 in Acute Stroke Treatment (TOAST) criteria. Stroke.

[20] Meschia JF, Barrett KM, Chukwudelunzu F, Brown WM, Case LD, Kissela BM, Brown RD, Brott TG, Olson TS, Rich SS, Silliman S, Worrall BB (2006). Interobserver agreement in the Trial of Org 10172 in Acute Stroke Treatment classification of stroke based on retrospective medical record review. J Stroke Cerebrovasc Dis.

[21] Nam HS, Cha MJ, Kim YD, Kim EH, Park E, Lee HS, Nam CM, Heo JH (2012). Use of a handheld, computerized device as a decision support tool for stroke classification. Eur J Neurol.

[22] Gordon DL, Bendixen BH, Adams HP, Clarke W, Kappelle LJ, Woolson RF (1993). Interphysician agreement in the diagnosis of subtypes of acute ischemic stroke: implications for clinical trials. The TOAST Investigators. Neurology.

[23] Wolf ME, Sauer T, Alonso A, Hennerici MG (2012). Comparison of the new ASCO classification with the TOAST classification in a population with acute ischemic stroke. J Neurol.

[24] Cotter PE, Belham M, Martin PJ (2012). Towards understanding the cause of stroke in young adults utilising a new stroke classification system (A-S-C-O). Cerebrovasc Dis.

[25] Chen N, Zhou M, Wang Y, Wang H, Yang M, Guo J, Yang X, Zheng H, Zhou D, He L (2013). Inter-rater reliability of the A-S-C-O classification system for ischemic stroke. J Clin Neurosci.

[26] Marnane M, Duggan CA, Sheehan OC, Merwick A, Hannon N, Curtin D, Harris D, Williams EB, Horgan G, Kyne L, McCormack PM, Duggan J, Moore A, Crispino-O'Connell G, Kelly PJ (2010). Stroke subtype classification to mechanism-specific and undetermined categories by TOAST, A-S-C-O, and causative classification system: direct comparison in the North Dublin population stroke study. Stroke.

[27] Arsava EM, Ballabio E, Benner T, Cole JW, Delgado-Martinez MP, Dichgans M, Fazekas F, Furie KL, Illoh K, Jood K, Kittner S, Lindgren AG, Majersik JJ, Macleod MJ, Meurer WJ, Montaner J, Olugbodi AA, Pasdar A, Redfors P, Schmidt R, Sharma P, Singhal AB, Sorensen AG, Sudlow C, Thijs V, Worrall BB, Rosand J, Ay H (2010). The Causative Classification of Stroke system: an international reliability and optimization study. Neurology.

[28] Ay H, Benner T, Arsava EM, Furie KL, Singhal AB, Jensen MB, Ayata C, Towfighi A, Smith EE, Chong JY, Koroshetz WJ, Sorensen AG (2007). A computerized algorithm for etiologic classification of ischemic stroke: the Causative Classification of Stroke System. Stroke.

[29] Quinn TJ, Dawson J, Walters MR, Lees KR (2009). Functional outcome measures in contemporary stroke trials. Int J Stroke.

[30] Quinn TJ, Dawson J, Walters MR, Lees KR (2009). Reliability of the modified Rankin Scale: a systematic review. Stroke.

[31] Wilson JTL, Hareendran A, Hendry A, Potter J, Bone I, Muir KW (2005). Reliability of the modified Rankin Scale across multiple raters: benefits of a structured interview. Stroke.

[32] Hanley DF, Lane K, McBee N, Ziai W, Tuhrim S, Lees KR, Dawson J, Gandhi D, Ullman N, Mould WA, Mayo SW, Mendelow AD, Gregson B, Butcher K, Vespa P, Wright DW, Kase CS, Carhuapoma JR, Keyl PM, Diener-West M, Muschelli J, Betz JF, Thompson CB, Sugar EA, Yenokyan G, Janis S, John S, Harnof S, Lopez GA, Aldrich EF, Harrigan MR, Ansari S, Jallo J, Caron J-L, LeDoux D, Adeoye O, Zuccarello M, Adams HP, Rosenblum M, Thompson RE, Awad IA (2017). Thrombolytic removal of intraventricular haemorrhage in treatment of severe stroke: results of the randomised, multicentre, multiregion, placebo-controlled CLEAR III trial. Lancet.

[33] van der Worp HB, Macleod MR, Bath PM, Demotes J, Durand-Zaleski I, Gebhardt B, Gluud C, Kollmar R, Krieger DW, Lees KR, Molina C, Montaner J, Roine RO, Petersson J, Staykov D, Szabo I, Wardlaw JM, Schwab S (2014). EuroHYP-1: European multicenter, randomized, phase III clinical trial of therapeutic hypothermia plus best medical treatment vs. best medical treatment alone for acute ischemic stroke. Int J Stroke.

[34] Fam MD, Hanley D, Stadnik A, Zeineddine HA, Girard R, Jesselson M, Cao Y, Money L, McBee N, Bistran-Hall AJ, Mould WA, Lane K, Camarata PJ, Zuccarello M, Awad IA (2017). Surgical performance in Minimally Invasive Surgery Plus Recombinant Tissue Plasminogen Activator for Intracerebral Hemorrhage Evacuation phase III clinical trial. Neurosurgery.

[35] Reinink H, de Jonge JC, Bath PM, van de Beek D, Berge E, Borregaard S, Ciccone A, Csiba L, Demotes J, Dippel DW, Kõrv J, Kurkowska-Jastrzebska I, Lees KR, Macleod MR, Ntaios G, Randall G, Thomalla G, van der Worp HB (2018). PRECIOUS: PREvention of Complications to Improve OUtcome in elderly patients with acute Stroke. Rationale and design of a randomised, open, phase III, clinical trial with blinded outcome assessment. Eur Stroke J.

[36] Duffy L, Gajree S, Langhorne P, Stott DJ, Quinn TJ (2013). Reliability (inter-rater agreement) of the Barthel Index for assessment of stroke survivors: systematic review and meta-analysis. Stroke.

[37] Connolly SJ, Ezekowitz MD, Yusuf S, Eikelboom J, Oldgren J, Parekh A, Pogue J, Reilly PA, Themeles E, Varrone J, Wang S, Alings M, Xavier D, Zhu J, Diaz R, Lewis BS, Darius H, Diener HC, Joyner CD, Wallentin L (2009). Dabigatran versus warfarin in patients with atrial fibrillation. N Engl J Med.

[38] Patel MR, Mahaffey KW, Garg J, Pan G, Singer DE, Hacke W, Breithardt G, Halperin JL, Hankey GJ, Piccini JP, Becker RC, Nessel CC, Paolini JF, Berkowitz SD, Fox KA, Califf RM (2011). Rivaroxaban versus warfarin in nonvalvular atrial fibrillation. N Engl J Med.

[39] Granger CB, Alexander JH, McMurray JJ, Lopes RD, Hylek EM, Hanna M, Al-Khalidi HR, Ansell J, Atar D, Avezum A, Bahit MC, Diaz R, Easton JD, Ezekowitz JA, Flaker G, Garcia D, Geraldes M, Gersh BJ, Golitsyn S, Goto S, Hermosillo AG, Hohnloser SH, Horowitz J, Mohan P, Jansky P, Lewis BS, Lopez-Sendon JL, Pais P, Parkhomenko A, Verheugt FW, Zhu J, Wallentin L (2011). Apixaban versus warfarin in patients with atrial fibrillation. N Engl J Med.

[40] Sanna T, Diener H-C, Passman RS, Di Lazzaro V, Bernstein RA, Morillo CA, Rymer MM, Thijs V, Rogers T, Beckers F, Lindborg K, Brachmann J (2014). Cryptogenic stroke and underlying atrial fibrillation. N Engl J Med.

[41] Gladstone DJ, Spring M, Dorian P, Panzov V, Thorpe KE, Hall J, Vaid H, O'Donnell M, Laupacis A, Côté R, Sharma M, Blakely JA, Shuaib A, Hachinski V, Coutts SB, Sahlas DJ, Teal P, Yip S, Spence JD, Buck B, Verreault S, Casaubon LK, Penn A, Selchen D, Jin A, Howse D, Mehdiratta M, Boyle K, Aviv R, Kapral MK, Mamdani M (2014). Atrial fibrillation in patients with cryptogenic stroke. N Engl J Med.

