# Supplementary material for: Stroke aetiological classification reliability and effect on trial sample size: systematic review, meta-analysis and statistical modelling
Source: Trials. 2019 Feb 8;20:107. doi: 10.1186/s13063-019-3222-x (PMC6368715; doi:10.1186/s13063-019-3222-x)
Supplement: Supplementary file 1 — Table S1. Search strategy. Table S2. Reporting quality and risk of bias assessment of the studies. Table S3. Baseline characteristics of the VISTA cohort used to analyse the effect of stroke classification on trial sample size. Figure S1. PRISMA 2009 flow diagram: literature search strategy. Figure S2. Forest plot describing inter-observer reliability (κ) across studies with different versions of TOAST. CI confidence interval. Figure S3. Forest plot describing inter-observer reliability (κ) across the studies for classic version of TOAST. CI confidence interval. Figure S4. Forest plot comparing inter-observer reliability (κ) across the studies for CCS subtypes. CI confidence interval. Figure S5. Forest plot describing inter-observer reliability (κ) across studies for CCS 5 subtype. CI confidence interval. Supplemental Results. Description of aggregate analysis using VISTA cohort. (DOCX 210 kb) [file 13063_2019_3222_MOESM1_ESM.docx]

**ADDITIONAL FILE 1**

Online-only Supplement for manuscript entitled:

**Stroke aetiological classification reliability and effect on trial sample size: systematic review, meta-analysis and statistical modelling**

**Authors:**

Azmil H. Abdul-Rahim, MD, MSc(StrokeMed)

David Alexander Dickie, PhD, MSc

Johann R. Selvarajah, PhD

Kennedy R Lees, MD

Terence J. Quinn, MD

*On behalf of VISTA collaborators*

**Supplemental Tables: 3**

**Supplemental Figures: 5**

**Supplemental Results:** Description of aggregate analysis using VISTA cohort.

**Additional file 1: Table S1.** Search strategy.

| **Databases searched**  EMBASE (OVID), MEDLINE (OVID), LILICAS (BIREME), CINAHL (EBSCO) and PUBMED |
| --- |

| **Key words for stroke:**  **String 1:**  cerebrovascular disorders/ or exp basal ganglia cerebrovascular disease/ or exp brain ischemia/ or exp carotid artery diseases/ or exp intracranial arterial diseases/ or exp intracranial arteriovenous malformations/ or exp intracranial embolism and thrombosis/ or exp intracranial hemorrhages/ or stroke/ or exp brain infarction/ or vasospasm, intracranial/ or vertebral artery dissection/  **String 2:**  (stroke or poststroke or post-stroke or cerebrovasc$ or brain vasc$ or cerebral vasc$ or cva$ or apoplex$ or SAH).tw.  **String 3:**  ((brain$ or cerebr$ or cerebell$ or intracran$ or intracerebral) adj5 (isch?emi$ or infarct$ or thrombo$ or emboli$ or occlus$)).tw.  **String 4:**  ((brain$ or cerebr$ or cerebell$ or intracerebral or intracranial or subarachnoid) adj5 (haemorrhage$ or hemorrhage$ or haematoma$ or hematoma$ or bleed$)).tw.  **String 5:**  hemiplegia/ or exp paresis/  **String 6:**  (hemipleg$ or hemipar$ or paresis or paretic).tw.  **or/String 1-6** | **Key words for reliability:**  Reliability or exp Reproducibility of Results or exp Reliability of Results or exp Diagnostic Error or exp Observer variation or exp Interobserver variability or exp Inter-observer variability or exp Interobserver variation or exp Inter-observer variation or exp Observer bias | **Key words for stroke classification system:**  (((Stroke data bank subtype classification or NINDS or The National Institute of Neurological Disorders) and Stroke) or Harvard Stroke Registry classification or Trial of ORG 10172 in Acute Stroke Treatment Subtype Classification or TOAST or Stop-Stroke Study TOAST or SSS-TOAST or The Causative Classification System or CCS or ASCO or ASCOD or GENIC or The Lausanne Stroke Registry or GEECV or Kim et al or Han et al or The Chinese ischemic stroke classification or CISS) |
| --- | --- | --- |
| or/String 1-6 **AND** Key words for stroke classification system  or/String 1-6 **AND** reliability **AND** Key words for stroke classification system | | |

**Table S2.** Reporting Quality and Risk of Bias assessment of the studies.

|  | Stroke aetiological classification system described | Subject population described | Rater population described | Sample size calculation described | The sampling method was described and appropriate | Blinding used and described | Assessment timing described | Reliability described with  95% CI |
| --- | --- | --- | --- | --- | --- | --- | --- | --- |
| *INTER-RATER reliability studies* | | | | | | | | |
| Arsava et al,2001 | yes | yes | yes | yes | yes | no | yes | yes |
| Ay et al, 2005 | yes | yes | yes | no | yes | no | yes | yes |
| Ay et al, 2007 | yes | yes | yes | no | yes | no | yes | yes |
| Chen et al, 2013 | yes | yes | yes | no | yes | yes | yes | yes |
| Fure et al, 2005 | yes | yes | yes | yes | yes | yes | yes | no |
| Goldstein et al, 2001 | yes | yes | yes | no | yes | no | yes | yes |
| Gordon et al, 1993 | yes | yes | yes | no | no | no | yes | no |
| Han et al, 2007 | yes | yes | yes | no | yes | no | yes | no |
| Marnane et al, 2010 | yes | no | yes | no | yes | no | yes | no |
| Meschia et al, 2006 | yes | yes | yes | no | yes | yes | yes | no |
| Nam et al, 2012 | yes | yes | yes | no | yes | no | yes | yes |
| Selvarajah et al,2009 | yes | yes | yes | no | yes | yes | yes | no |
| Wolf et al, 2012 | yes | Yes | yes | no | yes | yes | yes | no |
| *INTRA-RATER reliability studies* | | | | | | | | |
| Cotter et al, 2011 | yes | yes | yes | yes | yes | no | yes | no |

CI indicates confidence intervals.

**Table S3.** Baseline characteristics of the VISTA cohort used to analyse the effect of stroke classification on trial sample size.

| **Variable** | **Patients with cardioembolic stroke (n=521)** | **Patients *without* cardioemblic stroke (n=1545)** | **P-value** |
| --- | --- | --- | --- |
| Age, mean (SD), years | 74.4 (10.7) | 66.6 (13.2) | <.01 |
| Female | 268 (51.4) | 694 (44.9) | .01 |
| Prior antiplatelet treatment | 207 (39.7) | 1171 (75.8) | <.01 |
| Prior anticoagulant treatment | 314 (60.7) | 374 (24.2) | <.01 |
| Baseline NIHSS, median (IQR) | 11 (8) | 14 (8) | <.01 |
| Medical history |  |  |  |
| Myocardial infarction | 64 (12.3) | 167 (10.8) | 0.38 |
| Hypertension | 399 (76.6) | 1072 (69.4) | <.01 |
| Diabetes | 118 (22.7) | 333 (21.6) | 0.62 |
| Chronic heart failure | 96 (18.4) | 71 (4.6) | <.01 |
| Previous stroke | 134 (25.7) | 306 (19.8) | <.01 |
|  |  |  |  |
| Modified Rankin Scale at 90 days, median (IQR) | 3 (3) | 2 (3) | <.01 |

Abbreviations: NIHSS, National Institutes of Health Stroke Scale; IQR: interquartile range

Data are presented as number (percentage) unless otherwise indicated.

**Figure S1.** PRISMA 2009 flow diagram: Literature search strategy.

Additional articles identified through other sources

n= 719

Articles identified through database searching

n= 3936

Articles irrelevant

n= 2691

Articles after duplicates removed, n= 3030

**Identification**

Articles excluded

n= 260

Articles screened

n= 339

**Screening**

Full-text articles excluded, with reasons

n= 65^*^

Full-text articles assessed for eligibility

n= 79

**Eligibility**

Studies included in qualitative synthesis

n= 14

**Included**

Studies included in quantitative synthesis

(meta-analysis)

n=8

TOAST= 4

CCS= 4

^*^Conference articles

^*^Studies in children

^*^No inter-reliability

^*^No English articles

**Figure S2.** Forest plot describing interobserver reliability (κ) across studies with different versions of TOAST. CI indicates confidence intervals.


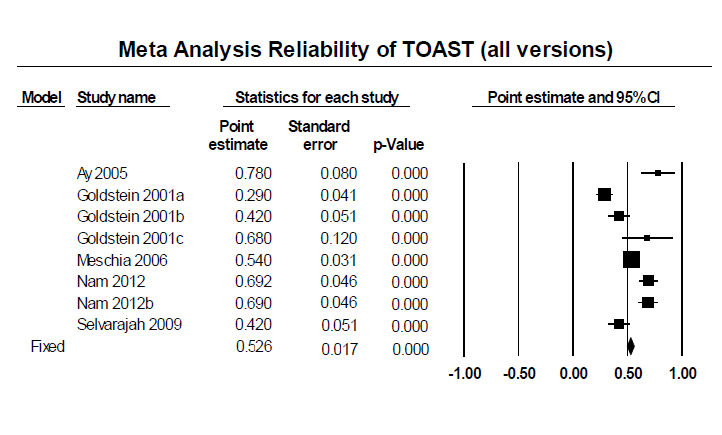


**Figure S3.** Forest plot describing interobserver reliability (κ) across the studies for classic version of TOAST. CI indicates confidence intervals.


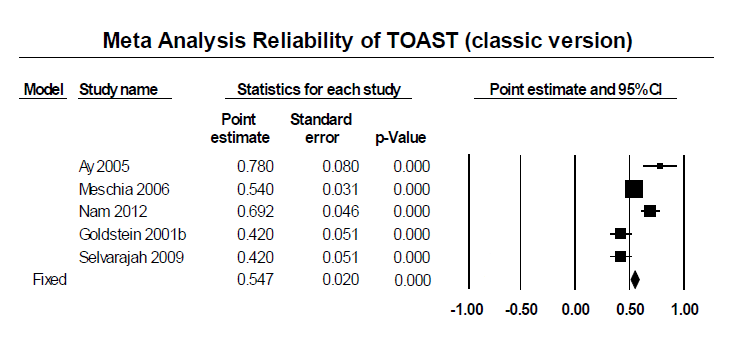


**Figure S4.** Forest plot comparing interobserver reliability (κ) across the studies for CCS subtypes. CI indicates confidence intervals.

**
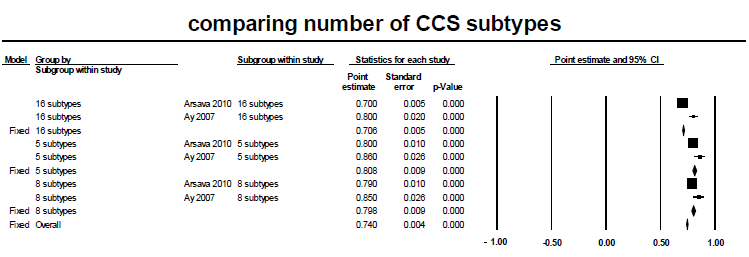
**

**Figure S5.** Forest plot describing interobserver reliability (κ) across studies for CCS 5 subtype. CI indicates confidence intervals.

**
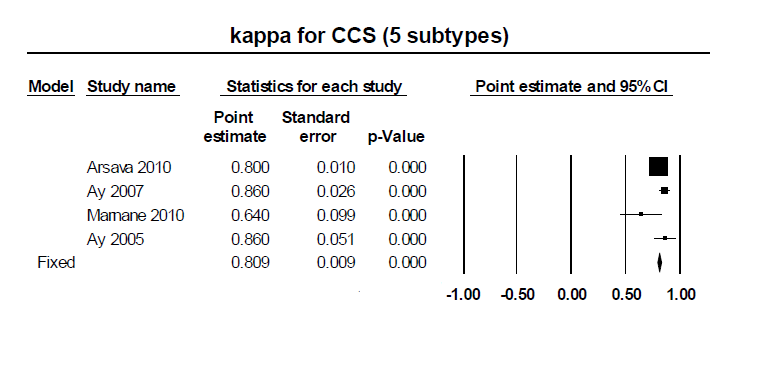
**

**Supplemental Results:** Description of aggregate analysis using VISTA cohort.

1. **Outcome: Death**

| *Total N=2066*  *Deceased N=338* | Cardioembolic stroke | Non-cardioembolic stroke |
| --- | --- | --- |
| Antiplatelet treatment (AP)  Deceased | 207 (10% of total N)  67 (32% of 207) | 1171 (57%)  141 (12%) |
| Anticoagulant (AC)  Deceased | 314 (15%)  67 (21%) | 374 (18%)  63 (17%) |

Note: Percentages for deceased patients are of N within each class.

**Sample size for death rate in cardioembolic stroke treated with anticoagulant versus antiplatelet**

From the proportions in the table above, sample size required per arm to detect a difference in death rate between AC (0.213 death rate) and AP (0.324 death rate) in AF is N=198 (power=0.8, alpha=0.05).

**Contamination of patients with non-CE stroke in a hypothetical trial of anticoagulant versus antiplatelet treatment in patients with CE stroke, via permutation testing for death rate**

The following data are from N=5000 permutations:

Death rate in AC arm contaminated with 5% non-CE stroke patients: 0.21

Death rate in AC arm contaminated with 20% non-CE stroke patients: 0.20

Death rate in AP arm contaminated with 5% non-CE stroke patients: 0.31

Death rate in AP arm contaminated with 20% non-CE stroke patients: 0.28

**Table A.** Table of death rates in AC versus AP arm and required sample size (in brackets)

| AP arm  AC arm | No contamination | 5% contamination | 20% contamination |
| --- | --- | --- | --- |
| No contamination | 0.21 versus 0.32 (198) | 0.21 versus 0.31 (237) | 0.21 versus 0.28 (466) |
| 5% contamination | 0.21 versus 0.32 (198) | 0.21 versus 0.31 (237) | 0.21 versus 0.28 (466) |
| 20% contamination | 0.20 versus 0.32 (165) | 0.20 versus 0.31 (193) | 0.20 versus 0.28 (352) |

Note: Sample sizes calculated with power=0.8, alpha=0.05.

AC: anticoagulant

AP: antiplatelet

1. **Outcome: recurrent stroke**

Subjects with recurrent stroke, n (proportion of subjects in that group):

AC in CE stroke =64 (0.20)

AC in non-CE stroke =63 (0.17)

AP in CE stroke =56 (0.27)

AP in non-CE stroke =165 (0.14)

**Sample size for recurrent stroke rate in cardioembolic stroke treated with anticoagulant versus antiplatelet treatment**

From the proportions in the table above, sample size required per arm to detect a difference in recurrent stroke between AC (0.20 recurrent stroke rate) and AP (0.27 recurrent stroke rate) in cardioembolic stroke is N=502 (power=0.8, alpha=0.05).

**Contamination of patients with non-CE stroke in a hypothetical trial of anticoagulant versus antiplatelet treatment in patients with CE stroke, via permutation testing for recurrent stroke**

The following data are from N=5000 permutations:

Recurrent stroke in AC arm contaminated with 5% non-CE stroke patients: 0.20

Recurrent stroke in AC arm contaminated with 20% non-CE stroke patients: 0.20

Recurrent stroke in AP arm contaminated with 5% non-CE stroke patients: 0.26

Recurrent stroke in AP arm contaminated with 20% non-CE stroke patients: 0.24

**Table B.** Table of recurrent stroke rates in AC versus AP arm and required sample size (in brackets)

| AP arm  AC arm | No contamination | 5% contamination | 20% contamination |
| --- | --- | --- | --- |
| No contamination | 0.20 versus 0.27 (502) | 0.20 versus 0.26 (630) | 0.20 versus 0.24 (1348) |
| 5% contamination | 0.20 versus 0.27 (484) | 0.20 versus 0.26 (605) | 0.20 versus 0.24 (1268) |
| 20% contamination | 0.20 versus 0.27 (409) | 0.20 versus 0.26 (502) | 0.20 versus 0.24 (973) |

Note: Sample sizes calculated with power=0.8, alpha=0.05.

AC: anticoagulant

AP: antiplatelet
